# miR-191: an emerging player in disease biology

**DOI:** 10.3389/fgene.2014.00099

**Published:** 2014-04-23

**Authors:** Neha Nagpal, Ritu Kulshreshtha

**Affiliations:** RNA-II Lab, Department of Biochemical Engineering and Biotechnology, Indian Institute of Technology DelhiNew Delhi, India

**Keywords:** miR-191, microRNA, cancer, disease, diagnosis, prognosis

## Abstract

Specific microRNAs have emerged as key players in disease biology by playing crucial role in disease development and progression. This review draws attention to one such microRNA, miR-191 that has been recently reported to be abnormally expressed in several cancers (>20) and various other diseases like diabetes-type 2, Crohn’ s, pulmonary hypertension, and Alzheimer’ s. It regulates important cellular processes such as cell proliferation, differentiation, apoptosis, and migration by targeting important transcription factors, chromatin remodelers, and cell cycle associated genes. Several studies have demonstrated it to be an excellent biomarker for cancer diagnosis and prognosis leading to two patents already in its kitty. In this first review we summarize the current knowledge of the regulation, functions and targets of miR-191 and discuss its potential as a promising disease biomarker and therapeutic target.

## INTRODUCTION

MicroRNAs are a class of small non-coding RNAs that function as post-transcriptional gene regulators (Bartel and Chen, 2004). Volinia et al. (2006) for the first time suggested the association of solid tumors with dysregulation of specific microRNAs. Since then miRNA-cancer link has extended beyond reports of abnormal miRNA levels in various cancers to miRNAs acting themselves as oncomiRs or tumor-suppressors. However, functional significance of majority of dysregulated miRNAs has not been elucidated. We have focused here on one such highly conserved miRNA, miR-191 that was found to be abnormally expressed in more than twenty different cancers and shown to be a major player in some of these. Further, altered expression of miR-191 has also been associated with various other diseases such as type 2 diabetes, neurodegenerative diseases, and more recently with innate immunity (Saba et al., 2008; Xu et al., 2009; Zampetaki et al., 2010). This review is timely to highlight the crucial role miR-191 plays in a multitude of diseases including cancer and also in normal development and differentiation processes. We start off with a small introduction to miR-191/425 cluster and its regulation followed by summarizing studies on functional dissection of miR-191 in various cancers. Overall, current understanding of miR-191 biology suggests it to be a promising novel target for cancer prognosis and therapy.

## miR-191/425 CLUSTER

miR-191 is expressed as part of the miR-191/425 cluster which is highly conserved in several metazoan species (miR-425 in 26 species, miR-191 in 30 species) suggesting it to be an important player in higher eukaryotes (Kiezun et al., 2012). First discovered in mouse, its expression was later confirmed in humans in a leukemia cell line (HL-60) and subsequently in 20 different human tissues (Lagos-Quintana et al., 2003; Kasashima et al., 2004). This cluster is located in the first intron of DALRD3 gene on human chromosome 3 (3p21.31) and codes for four mature miRNAs – miR-191-5p, miR-191-3p, miR-425-5p, and miR-425-3p. It forms a sense antisense transcript pair with DALRD3, NDUFA3, and WDR6 mRNAs; however, the functional impact of this association has not been experimentally evaluated (Griffiths-Jones et al., 2006). miR-191 tends to be coexpressed with the DALRD3 and NDUFAF3 transcripts. miR-191 resides 381 nucleotides upstream of miR-425 and notably shows much higher expression or cloning frequency as compared to miR-425 in all the tissues studied so far (Altuvia et al., 2005; Di Leva et al., 2013). This is in compliance with recent reports showing that huge variations in expression levels of miRNA members may exist within the same cluster (Vaz et al., 2010). Indeed, Croce and Kulshreshthas group have shown that both miR-191 and miR-425 promote cell proliferation in breast cancer exhibiting overlapping functions, but a previous study shows that miR-191 and not miR-425 promotes erythroid enucleation (Zhang et al., 2011; Di Leva et al., 2013; Nagpal et al., 2013). Therefore, with very limited data, we assume that miR-191 and miR-425 may exhibit specific or overlapping functions depending on the tissue and conditions.

## miR-191 IN DEVELOPMENT AND DIFFERENTIATION

The first report that miR-191 is developmentally regulated came from the observation that significantly high levels of miR-191 were present in (postmortem) human prefrontal cortex specimens of individuals older than 41 years than those under 15 years. It was suggested that miR-191 mediated downregulation of brain-derived neurotrophic factor (BDNF; predicted miR-191 target) may contribute to the cortical development (Mellios et al., 2008). miR-191 is also one of the major miRNA expressed in rat neurons and its expression increases ~2–5 fold with time in rat cortical cultures suggestive of its role in neuronal development (Kim et al., 2004).

Developmental association of miR-191 was further extended to senescence since its overexpression was shown to promote replicative senescence and inhibit proliferation in primary human keratinocytes (Lena et al., 2012). Role of miR-191 in regulation of circadian rhythm was also suggested as it was shown to exhibit a circadian pattern of expression in mouse liver and showed inverse correlation to Bma-1 (circadian transcription factor) levels (Na et al., 2009).

The connection of miR-191 and differentiation was also explored recently. miR-191 was found to be significantly downregulated during terminal erythroid differentiation (CFU-E to the Ter119+ stage) while its overexpression inhibited erythroid enucleation and chromatin condensation suggesting that miR-191 plays a crucial role in erythropoiesis (Zhang et al., 2011). Levels of miR-191 were also found to be differentially expressed in monocytes vs. monocytes derived dendritic cells (Cekaite et al., 2010). Induced miR-191 levels were observed in osteoblast like cells following treatment with Bio-Oss (an organic bovine bone). As Bio-Oss plays an important role in bone regeneration thus miR-191 may be an important mediator for the same (Palmieri et al., 2010). Therefore, all these findings suggest that miR-191 plays an important role in cellular differentiation and development.

## miR-191 AND CANCER CONNECTION

miR-191 and cancer abnormalities have been reported in more than twenty different malignancies making the miR-191 a ubiquitously notorious miRNA like miR-21 or miR-155. Overexpression of miR-191 in 16 different cancer types [breast (female), colon, lung, liver, prostate, pancreas, stomach, ovarian cancer, pituitary adenoma, esophageal squamous carcinoma, oral squamous carcinoma, osteosarcoma, B-ALL, bladder, anaplastic large cell lymphoma, and acute myeloid leukemia (AML)] classifies it largely as an oncogenic miRNA. However, in six other cancer types (severe medulloblastomas, retinoblastoma, thyroid follicular tumor, male breast cancer, CALL, and melanoma) its levels are known to be downregulated (Volinia et al., 2006; Xi et al., 2006; Garzon et al., 2008; Fassan et al., 2009; Ferretti et al., 2009; Fulci et al., 2009; Hui et al., 2009; Kent et al., 2009; Caramuta et al., 2010; Elyakim et al., 2010; Patnaik et al., 2010; Shen et al., 2010; Colamaio et al., 2011; Duan et al., 2011; He et al., 2011; Leite et al., 2011, 2013; Li et al., 2011; Shi et al., 2011; Xu et al., 2011; Inoue et al., 2012; McEvoy et al., 2012; Poliseno et al., 2012; Di Leva et al., 2013; Gombos et al., 2013; Liu et al., 2013a; Nagpal et al., 2013; Zhou et al., 2013; Scheffer et al., 2014). The nature of miR-191 deregulation, its functions and targets reported so far in various cancers are shown in **Table 1**.

**Table 1:** Summary of disease subtypes reported to have aberrantly expressed miR-191 along with the summary on cellular effects/disease prognosis and experimentally validated target transcripts.

Type of disease	Expression	Biological process affected due to aberrantly expressed miR-191	Experimentally validated targets responsible for function and regulation	Reference
**Cancer subtypes with aberrantly expressed miR-191**
Acute myloid leukemia	Overexpressed in AML cohort of patients	Poor overall survival	–	Garzon et al. (2008)
Acute lymphoblastic leukemia	–	Discriminate B-lineage of ALL	–	Fulci et al. (2009)
Childhood acute lymphoblastic leukemia	Downregulated in pediatric ALL patients	Associated with disease prognosis	–	Xu et al. (2011), McEvoy et al. (2012)
Anaplastic large cell lymphoma	Upregulated in ALK(-) ALCL compared to that of PLCL	Can serve as diagnostic tool for disease characterization	–	Liu et al. (2013a), Ferretti et al. (2009)
Female breast cancer	Overexpressed in breast cancer tissues and cell lines	Critical mediator of ER-mediated cell proliferation. Can serve as a biomarker for early detection of breast cancer	SATB1, CDK6, BDNF, EGR1, CCND2,ESR1, ESR2	Iorio et al. (2005), Hui et al. (2009), Nagpal et al. (2013), Di Leva et al. (2013), Mar-Aguilar et al. (2013a,b)
Male breast cancer	Downregulated in male breast cancer tissues	May be involved in the development of the disease	–	Fassan et al. (2009)
Colorectal cancer	Overexpressed in colorectal tissue samples from patients and colon cancer cell lines	May be associated with the development of the disease	TIMP3	Zhou et al. (2010), Xi et al. (2006), Volinia et al. (2006), Monzo et al. (2008), Qin et al. (2014)
Gastric carcinoma	Upregulated in Gastric carcinoma tissues and cell lines	Promotes cell growth and suppresses apoptosis	NDST1	Li et al. (2011), Shi et al. (2011), Volinia et al. (2006)
Melanoma/malignant melanoma	Downregulated in the melanoma tumors	Poor melanoma specific survival	–	Poliseno et al. (2012), Caramuta et al. (2010)
Hepatocellular carcinoma	Overexpressed in HCC tissues	Promotes EMT, enhances cell proliferation and tumor growth	TIMP3, TMC7, SOX4,IL1A	He et al. (2011), Elyakim et al. (2010)
Pancreatic adenocarcinoma	Overexpressed in cyst fluid of pancreatic adenomas of the patients	Potential biomarker for classification of cystic lesions of the pancreas	–	Kent et al. (2009), Volinia et al. (2006)
Thyroid follicular tumors	Downregulated in follicular adenoma	Cell cycle progression	CDK6	Colamaio et al. (2011)
Retinoblastoma	Significantly downregulated in retinoblastoma compared to that of fetal retinae	Inactivation of p53 activity	MDM4-C	McEvoy et al. (2012)
Prostate cancer	Downregulated in prostrate carcinoma of patients	Prostrate cancer progression and metastasis	–	Leite et al. (2011),^2013^, Volinia et al. (2006)
Ovarian cancer	Upregulated in ovarian cancer patients	Ovarian cancer progression and tumor related death	MDM4-C	Shen et al. (2010), Wynendaele et al. (2010)
Lung cancer	Overexpressed in lung cancer cell lines	No effect on the phenotype	–	Volinia et al. (2006), Patnaik et al. (2010)
Osteosarcoma	Upregulated in osteosarcoma cell lines	–	–	Duan et al. (2011)
Oral squamous cell carcinoma	Upregualted in OSCC tissues	Helpful in disease diagnosis and prognosis	–	Gombos et al. (2013)
Esophageal squamous carcinoma	–	Can be used as a reference miRNA in ESCC	MDM4-C	Zhou et al. (2013), Saad et al. (2013)
Bladder cancer	Upregulated in bladder cancer patients	Helpful in disease diagnosis	–	Scheffer et al. (2014)
**Other diseases with aberrantly expressed miR-191**
Type 2 diabetes	Downregulated in type 2 diabetes patients	Impaired peripheral angiogenic signaling	–	Zampetaki et al. (2010)
Chronic fatigue syndrome	Downregulated in peripheral blood of CFS patients	Apoptosis, cell cycle, development and immune function	–	Brenu et al. (2012)
Pulmonary hypertension	Upregulated in PH subjects	Potential biomarker for early detection of disease	–	Wei et al. (2013)
Neurodegenerative diseases	Upregulated in prion induced mouse brain	Regulated KROX family of proteins	EGR1	Saba et al. (2008)
Alzheimer’ s disease	Downregulated (3–5 fold) in patients suffering from the disease	Biomarker for prediction of the disease	–	Kumar et al. (2013)
Preeclampsia	Upregulated in PE placenta	Can be useful in disease pathogenesis	–	Choi et al. (2013)
Idiopathic nephrotic syndrome	Highly upregulated in NS children vs. control, significant reduced level in patients after therapy	Can be helpful in disease progression and therapy	–	Luo et al. (2013)
Keratinocyte senescence	Downregulated in keratinocyte neonatal cell lines	Proliferation inhibitor and senescence induction	CDK6, SATB1	Lena et al. (2012)
Inflammatory bowel disease	Upregulated in the blood of the patients of Crohn’ s disease	Unique diagnosis of Crohn’ s disease and ulcerative colitis	–	Paraskevi et al. (2012), Lin et al. (2013)
Erythroblast enucleation of mouse	Downregulated in the erythroblasts of mice	Chromatin condensation and erythroblast enucleation	RIOK3, MXI1	Zhang et al. (2011)

### miR-191 AS A BIOMARKER FOR CANCER DIAGNOSIS AND PROGNOSIS

Since miR-191 is one of the highly expressed and stable miRNA in human serum or saliva, it shows potential of being used as non-invasive biomarker in human subjects (Patel et al., 2011). A new method for the disease characterization based upon blood circulating miRNAs was proposed recently. It involved both miR-191 and miR-425 as part of a unique combination of 12 miRNAs that could serve as potential biomarkers to distinguish disease/cancer from healthy controls in 10 out of 13 diseases (*p* = 0.046; Taguchi and Murakami, 2013). Zhang et al. (2012) recently filed a patent involving a group of 7 miRNAs including miR-191 that serve as markers for prediction, diagnosis, and progression of pancreatic cancer. Similarly, miR-191 (along with miR-182 and miR-199a) based methods for diagnosis and prognosis of AML have been recently patented (Croce, 2013). Higher expression of miR-191 was found to be significantly associated with poor overall survival (OS) and event free survival in adult AML patients (Garzon et al., 2008). Also, in colorectal cancer patients, higher miR-191 expression has been shown to be associated with clinical stage, metastasis and tumor invasion (Xi et al., 2006). In contrast, reduced miR-191 levels have been reported to be associated with bad prognosis or short OS or disease relapse in melanoma, pediatric AML or male smoker squamous cell carcinoma (SCC) patients (Mueller et al., 2009; Zhang et al., 2009; Caramuta et al., 2010; Landi et al., 2010; Xu et al., 2011).

Apart from miR-191 alone as a prognostic marker, the miR-146b/miR-191 ratio has also been reported as a negative prognostic indicator in lung SCC patients (0.29+0.25; Ernest et al., 2012). Likewise, Mar-Aguilar et al. (2013a) have shown that combined expression profile of two miRNAs (miR-125b/miR-191 and miR-21/miR-191) is more specific in discriminating between breast cancer and non-tumor tissue (Mar-Aguilar et al., 2013a). Interestingly, presence of genetic variants in miR-191 precursor has been linked to genetic predisposition to familial ovarian cancer and could therefore serve as a diagnostic marker (Duan et al., 2011). Additionally, epigenetic regulation (hypomethylation) of miR-191 locus has been associated with poor prognosis in hepatocellular carcinoma (HCC; He et al., 2011). Hence, quantification of miR-191/its precursor variants/methylation patterns of its promoter using tissue/blood samples may emerge as a novel method for cancer diagnosis and prognosis.

### miR-191 AS A REGULATOR OF VARIOUS HALLMARKS OF CANCER

While there are ample reports on abnormal miR-191 levels in various cancers, the knowledge about its functional impact is quite limited. Studies in breast, colon, gastric, and hepatic cancer cell lines suggest miR-191 to be an oncomiR (Xi et al., 2006; Elyakim et al., 2010; Shi et al., 2011; Di Leva et al., 2013; Nagpal et al., 2013). miR-191 was shown to be involved in regulation of cell proliferation, apoptosis, and epithelial mesenchymal transition in HCC (Elyakim et al., 2010; He et al., 2011). In colon cancer, anti-miR-191 was shown to attenuate the invasiveness, suppress proliferation and induce apoptosis and in gastric cancer, miR-191 overexpression was shown to enhance cell proliferation and reduce apoptosis (Shi et al., 2011; Qin et al., 2014). However, functional analysis of miR-191 levels in pancreatic ductal carcinoma cells showed that although it is responsible for maintaining the transformed stage but cell proliferation is not affected (Kent et al., 2009). Recently, Nagpal et al. (2013) showed that miR-191 is responsible for promotion of various hallmarks of cancer (proliferation, metastasis, stress resistance) in ER+ breast cancer (MCF7) cells, while Di Leva et al. (2013) demonstrated that miR-191/425 cluster is responsible for reduced proliferation, tumorigenesis, and metastasis in aggressive ER- breast cancer cells (MDA-MB-231; Di Leva et al., 2013; Nagpal et al., 2013). This dual effect of miR-191 could be due to the fact that miR-191 is an ER regulated miRNA that functions as a critical mediator of estrogen mediated cell proliferation. miR-191 has been shown to regulate cell cycle progression in breast cancer by promoting G1/S and G2/M transitions as the cells overexpressing miR-191 entered S-phase much faster with drastic reduction in G0/G1 phase.

In contrast, miR-191 has been projected as a tumor-suppressor microRNA that leads to reduced growth and migration in thyroid follicular carcinoma (Colamaio et al., 2011). Notably, though miR-191 was shown to be differentially expressed (significantly upregulated in both squamous and non-squamous NSCLC, *p* < 1e-07) in lung cancer, it was shown to not alter cell cycle, proliferation or chemosensitivity of lung cancer cell lines (Volinia et al., 2006; Yanaihara et al., 2006; Patnaik et al., 2010).

### miR-191 AS A THERAPEUTIC TARGET IN CANCER

Till date only two groups have performed mouse xenograft assays to test miR-191 activity *in vivo* evaluating its therapeutic potential and the results are certainly encouraging. Elyakim et al. (2010) generated an orthotopic human liver tumor xenografts in nude mice and administered 2-*O*-metoxyethyl (MOE) anti-miR-191 through repeated intraperitoneal injections. A statistically significant reduction in tumor mass was observed without toxicity by the end of 40 days (Elyakim et al., 2010). Similarly, a subcutaneous transplantation of the anti-miR-191 transfected ER-alpha positive MCF7 or ZR-75-1 breast cancer cells in nude mice resulted in a 50% reduction in tumor mass. The effect of miR-191 on metastasis was also studied in aggressive breast cancer. Injection of miR-191 overexpressing ER negative breast cancer cells (MDA-MB-231) in NOD-SCID mice led to a significant reduction in a number of micrometastasis to lungs of mice as compared to the control tumor cells (Di Leva et al., 2013). Thus, regulated expression of miR-191 may serve as potential therapeutic treatment of the disease.

### REGULATION OF miR-191 EXPRESSION IN CANCER

Despite well documented miR-191 and cancer links, very little is known about the factors that lead to its deregulation in various tumor cells. In breast cancer, two independent groups demonstrated that estrogen and tumor microenvironment play a major role in regulation of miR-191 (Di Leva et al., 2013; Nagpal et al., 2013). The promoter of miR-191 bears estrogen response elements which show dynamic binding of ER-alpha and ER-beta transcription factors in response to estrogen treatment (Nagpal et al., 2013). The levels of both miR-191 and miR-425 have been reported to be estrogen inducible (Di Leva et al., 2013; Nagpal et al., 2013). Tumor microenvironment (hypoxia and nutrient deprivation) has also been reported to strongly induce miR-191 in breast cancer (Nagpal et al., 2013). A report by Xi et al. (2006) suggests that the loss of p53 may be the reason behind higher expression (median 1.4-fold, *p* 0.0264) of miR-191 in colorectal tumor. Another study shows that the gain in expression of MDM4, a miR-191 target gene, in retinoblastoma leads to inactivation of p53 activity (McEvoy et al., 2012). Interestingly, the hypomethylation of CpG islands in miR-191 promoter has been linked to higher expression of miR-191 in HCC (He et al., 2011). While in melanoma, both, the genomic loss of the miR-191 locus and downregulation of miR-191 by a transcription factor, SNAIL have been shown as the reasons for low miR-191 levels (Mueller et al., 2009; Poliseno et al., 2012). To conclude, transcription factors, tumor microenvironment and epigenetic mechanisms play major role in regulation of miR-191 in various cancers.

Several recent reports suggest that miR-191 levels are also responsive to chemical drugs and radiation treatments. Chaudhry et al. (2013) have shown miR-191 induction in response to X-ray treatment in human lymphoblast cells (TK6 cell line). However, miR-191 was shown to be rather downregulated in two colon cancer cell lines HCT116 and HCT-8 when treated with chemotherapeutic drugs (5-Fluorouracil and Oxaliplatin; Zhou et al., 2010). Thus, the effects on miR-191 levels may vary depending on the stress or tissue/cell type. Work in our lab shows that miR-191 overexpressing breast cancer cells show increased survival in response to chemotherapeutic drug or hypoxia treatment (Nagpal et al., 2013). Altogether these reports suggest prospects of miR-191 being a target for studying chemotherapeutic interference (Zhou et al., 2010).

Interestingly, carcinogens too have been reported to mediate miR-191 regulation. Davidson et al. (2009) showed that AOM (carcinogen) treated rats show downregulation of miR-191. Conversely, feeding rats with fish oil rich in polyunsaturated fatty acids (*n* = 3 PUFA; a chemoprotective agent) prevented AOM mediated downregulation of miR-191 and tumor formation (Davidson et al., 2009). Environmental carcinogens also play a major role in regulation of miR-191, for example, cigarette smoke and TCDD (a dioxin family carcinogen) was shown to regulate miR-191 levels in rat lungs and hepatic cancer, respectively (Izzotti et al., 2009; Elyakim et al., 2010). Thus, these data provide evidence for hormonal, environmental and dietary regulation of miR-191.

## miR-191 IN DISEASES OTHER THAN CANCER

Association of miR-191 is not only limited to various cancers but its irregular expression and functional abnormalities have been reported in a variety of other diseases as well (**Table 1**). The functional impact of miR-191 is still unknown in these diseases but it does show potential as a diagnostic or prognostic marker for some of them. Recently Lin et al. (2013) reported that miR-191 may be a diagnostic biomarker candidate that could help to distinguish between the Crohn’s disease (CD) and ulcerative colitis (UC; Lin et al., 2013). Further, as the two disease types differ in their associated T- and B- cells, miR-191 may be involved in regulation of innate and adaptive immunity as well (Paraskevi et al., 2012). Similarly, miR-191 was shown to be a potential biomarker for early detection and severity of pulmonary hypertension (Wei et al., 2013). Remarkably, miR-191-5p was shown to be one of the best biomarker candidates to predict the Alzheimer’s disease with >95% accuracy (Kumar et al., 2013). miR-191 was also found to be significantly induced (3.79-fold, *p* < 0.0001) in nephrotic syndrome (NS) children making it a potential diagnostic marker for pediatric NS. Interestingly, NS patients under remission have significantly reduced level of miR-191 suggestive of its association with disease progression and therapy (Luo et al., 2013). Overall, miR-191 seems to be closely associated with the pathogenesis of diverse diseases and may also be involved in innate immune responses. A compiled list of diseases having an association with miR-191 is given in **Table 1**.

## miR-191 TARGETS

A number of miR-191 targets have been validated and characterized functionally. Its targets range from chromatin remodelers to transcription factors to cell cycle regulators. A detailed analysis of the validated potential targets of miR-191 is given below:

### TRANSCRIPTION FACTORS AND CHROMATIN REGULATORS

#### SATB1 (special AT-rich sequence-binding protein-1)

Special AT-rich sequence-binding protein-1 is a global chromatin remodeler and transcription factor that is known to be overexpressed in several cancers and also associated with aggressive phenotype in colorectal and breast cancer (Cai et al., 2006; Han et al., 2008; Meng et al., 2012). miR-191 mediated downregulation of SATB1 has been linked to gain/loss of epithelial/mesenchymal markers in aggressive breast cancer and enhanced cell proliferation and migration in hormone dependent breast cancer (Di Leva et al., 2013; Nagpal et al., 2013). The miR-191/SATB1 functional link has been proposed to be helpful for prognosis and therapeutics of breast cancer (Nagpal et al., 2013). Additionally, suppression of SATB1 was demonstrated to be linked to cell senescence, as its suppression by miR-191 led to enhanced senescence in keratinocytes (Lena et al., 2012).

#### RIOK3 (RIO kinase 3) and MXI1 (max-interacting protein 1)

RIO kinase 3 and max-interacting protein 1 are reported to be negatively regulated by miR-191 and play essential role in chromatin condensation and enucleation during mouse erythroid differentiation (Zhang et al., 2011).

#### EGR1 (early growth response protein 1)

Early growth response protein 1 is a zinc finger transcription factor mainly involved in cell growth, development and stem cell homeostasis (Yan et al., 2000). It was demonstrated to be an estrogen responsive miR-191 target, having regulatory role in cell proliferation in ER-alpha positive breast cancer cells. Additionally, it was reported to be downregulated by miR-191 in scrapie infection, a prion disease (Saba et al., 2008; Di Leva et al., 2013).

#### SOX4 SRY (sex determining region Y)-box 4

Sex determining region Y-box 4 is a transcriptional factor found to be upregulated in various cancers (Rhodes et al., 2004). It is involved in regulation of development, differentiation, proliferation, apoptosis, and more recently epithelial-mesenchymal-transition (Pan et al., 2009). It was shown to be a novel target of miR-191 in HCC (Elyakim et al., 2010). Thus, downregulation of SOX4 by miR-191 may affect chromatin architecture and crucial cellular processes.

#### MDM4 (Mdm4 p53 binding protein homolog)

Mdm4 p53 binding protein homolog is a negative regulator of transcription factor, p53 and is found to be overexpressed in 17% of all cancers (Liu et al., 2012). miR-191 is reported to regulate cancer progression and chemosensitivity in ovarian cancer by modulating the level of MDM4 through binding with an illegitimate site (rs4245739) within the 3′ UTR (Wynendaele et al., 2010). Recently, the same SNP (rs4245739) was investigated and found to be a risk factor for esophageal SCC and breast cancer (Zhou et al., 2013). The MDM4 rs4245739 SNP alone or in combination with the p53 variant Arg72Pro has been shown to be responsible for attenuating breast cancer risk in Chinese population (Liu et al., 2013b).

### CELL CYCLE REGULATORS

#### CDK6 (cyclin-dependent kinase 6)

Cyclin-dependent kinase 6 is a protein kinase mainly associated with cell cycle progression and differentiation (Robker and Richards, 1998; Depamphilis et al., 2012). It has been reported as a bona fide target of miR-191 in aggressive and hormone receptor positive breast cancer and thyroid and follicular carcinoma (Colamaio et al., 2011; Di Leva et al., 2013; Nagpal et al., 2013). Additionally, miR-191 mediated downregulation of CDK6 has been reported to trigger senescence in keratinocytes (Lena et al., 2012).

#### CCND2 (cyclin D2)

Cyclin D2 is a cell cycle regulatory protein involved in estrogen mediated cell proliferation (Robker and Richards, 1998). It was recently established as a target of miR-191 and its downregulation led to suppression of PI3/AKT pathway thereby leading to reduced cell proliferation in aggressive breast cancer (Di Leva et al., 2013).

### OTHERS

#### NDST1 (N-deacetylase/N-sulfotransferase1)

*N*-deacetylase/*N*-sulfotransferase1 is a key enzyme involved in the biosynthesis of heparan sulfate chains and is responsible for maintenance of physiological functions (Sheng et al., 2011). It regulates binding of FGF to mammary epithelial cells leading to branching morphogenesis (Crawford et al., 2010). It was shown to be negatively regulated by miR-191 and its downregulation has suppressive effect on cell proliferation in gastric carcinoma (Shi et al., 2011).

#### BDNF (brain-derived neurotrophic factor)

Brain-derived neurotrophic factor is a neurotrophin essential for differentiation, survival, and synaptic plasticity of CNS (Numakawa et al., 2011). Recently our group has shown it to be a target of miR-191 in ER positive breast cancer (Nagpal et al., 2013).

#### TIMP3 (tissue inhibitor of metalloprotease 3)

Tissue inhibitor of metalloprotease 3 is a proapoptotic protein whose expression is negatively correlated with cell growth and invasiveness (Kashiwagi et al., 2001). It is a direct miR-191 target in colorectal carcinoma and its targeted downregulation is associated with enhanced invasiveness of the disease (Qin et al., 2014).

**FIGURE 1:**
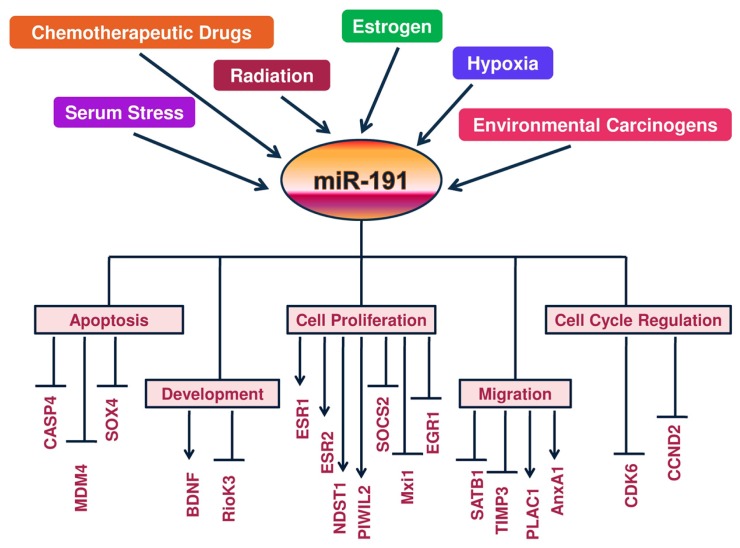
**A Schematic view of regulation of miR-191 and its targets: a molecular model is presented whereby hormones (estrogen), tumor microenvironment (hypoxia and serum stress) and DNA damage signals (chemodrugs, radiation and carcinogens) regulate miR-191 levels in the cancer cells.** In response, miR-191 controls the expression of a number of genes involved in a variety of cellular processes (ranging from cell proliferation to apoptosis to cell migration) to modulate the development of cancer. Overall, miR-191 integrates multiple pathogenic signals to regulate cancer phenotype.

An overview of miR-191 regulatory components along with its functional impact on direct and indirect targets is given in **Figure 1**.

## CONCLUDING REMARKS AND FUTURE PERSPECTIVES

Our review brings to limelight an important player in cancer biology that has been shown to have profound effects on various hallmarks of cancer. Due to its ubiquitous role in various biological processes, the interest in miRNA-191 is likely to dramatically increase in coming years, especially in cancer. Though various studies document miR-191 association with cancer progression, but its link to cancer initiation has not yet been elucidated. However, since carcinogens have been shown to regulate the levels of miR-191, it may be involved in tumor initiation process as well.

Considering the widespread association of miR-191 to several cancer types and recent studies documenting that its inhibition leads to reversal of cancer phenotype, it is tempting to speculate that miR-191 may qualify as a candidate for “oncogene addition.” However, the validation of this concept warrants careful and extensive experimentation using genetically engineered mouse models of miR-191. These models may also throw light on the role of miR-191 in various stages of tumor development. Nevertheless, based on miR-191-lung cancer studies, miR-191 may not necessarily functionally contribute to aggression of all cancer/diseases where its levels are abnormally expressed (Patnaik et al., 2010).

Interestingly, several types of abnormalities have been linked to miR-191 in cancer. These include but may not be limited to aberrant mature miR-191 levels, hypomethylated miR-191 promoter, mutations in miR-191 target site or loss of genomic locus (Caramuta et al., 2010; He et al., 2011). However, there are very few reports depicting the basis behind its altered expression in disease states compared to that of normal. Till date transcription factors, tumor microenvironment and epigenetic regulation have been identified as the major players responsible for aberrant expression of miR-191 in various disease states (He et al., 2011; Di Leva et al., 2013; Nagpal et al., 2013). A strong estrogen and hypoxic regulation of miR-191 have been shown to regulate key pathways in breast cancer (Di Leva et al., 2013; Nagpal et al., 2013). In light of recent reports of interdependence of estrogen and hypoxia pathways, it would be interesting to know if miR-191 functions to connect the two pathways. Further, considering miR-191 to be stress inducible and its reported effects on apoptosis, it would be interesting to see if p53 is directly involved in its regulation.

Owing to highly consistent and stable expression of miR-191 and the ability to detect it in human saliva or serum, miR-191 emerges as a strong candidate for non-invasive disease diagnostics (Patel et al., 2011). A strong association between miR-191 levels and disease prognosis/survival laid basis for two recent patents featuring miR-191 based diagnosis and prognosis in AML and pancreatic cancer (Zhang et al., 2012; Croce, 2013). It is likely that miR-191 may emerge as an independent or a co-prognostic marker in other cancers as well. Use of disease specific mouse models, conditional transgenic as well as knockouts are required to elucidate its functional role *in vivo* and verify its therapeutic potential, both of which remain largely unexplored till now. More novel approaches such as combination of Argonaute protein immunoprecipitation, deep sequencing, or proteomic profiling approaches are required to identify its potential bona fide targets responsible for its functional impact. The fact that miR-191 may directly enhance target transcript levels is striking and needs further elucidation of the mechanism. In particular a combination of phenotypic, transcriptomic, proteomic, and metabolomic changes on manipulation of miR-191 levels in cell line or mouse models will provide a complete understanding of miR-191 functions. To conclude, achieving ways to regulate the level of this particular microRNA in patients (in a safe and consistent manner) might help to fight the underlying disease.

## Conflict of Interest Statement

The authors declare that the research was conducted in the absence of any commercial or financial relationships that could be construed as a potential conflict of interest.
